# Longitudinal Tumor, Vascular, and Immune Cell Response in Two Rat Prostate Carcinomas After Isoeffective Photon, Proton, and Carbon Ion Irradiation: Impact of Linear Energy Transfer, Dose Level, and Hypoxia

**DOI:** 10.3390/cancers18162653

**Published:** 2026-08-17

**Authors:** Michaela Schmitt, Ina Kurth, Christin Glowa, Manuela Dittrich, Rosemarie Euler-Lange, Stephan Brons, Peter Peschke, Christian P. Karger

**Affiliations:** 1 Division of Medical Physics in Radiation Oncology, German Cancer Research Center (DKFZ), 69120 Heidelberg, Germany; 2 Faculty of Biosciences, Heidelberg University, 69120 Heidelberg, Germany; 3 Heidelberg Institute for Radiation Oncology (HIRO) and National Center for Radiation Research in Oncology (NCRO), 69120 Heidelberg, Germany; ina.kurth@dkfz.de (I.K.); m.dittrich@dkfz.de (M.D.); r.lange@dkfz.de (R.E.-L.); stephan.brons@med.uni-heidelberg.de (S.B.); 4 Division of Radiooncology/Radiobiology, German Cancer Research Center (DKFZ), 69120 Heidelberg, Germany; 5 Department of Radiation Oncology and Radiotherapy, University Hospital Heidelberg, 69120 Heidelberg, Germany; 6 Heidelberg Ion Beam Therapy Center (HIT), 69120 Heidelberg, Germany

**Keywords:** carbon ion radiotherapy, proton therapy, photon radiotherapy, prostate cancer, tumor microenvironment, hypoxia, histopathology, macrophages, T cells

## Abstract

**Background/Objectives**: High linear energy transfer (LET) carbon ions achieved more effective and biologically robust tumor control than photons in preclinical prostate cancer models; however, the longitudinal development of histopathological parameters remains insufficiently characterized, limiting the selection of the optimal treatment modality in patients. This study analyzed the temporal histological patterns after isoeffective photon, proton, and carbon ion irradiations. **Methods**: Two Dunning R3327 prostate carcinoma sublines (H, HI) grown subcutaneously in male Copenhagen rats received single-fraction isoeffective curative photon or carbon ion doses. For HI-tumors, the effectiveness of isoeffective curative proton doses and isoeffective subcurative photon and carbon ion doses was additionally investigated. Tumors were collected prior and up to 3 weeks after irradiation and processed for quantitative histology of proliferation (BrdU), DNA damage (γH2AX), hypoxia (pimonidazole), vascular (CD31), and immune cell (CD3, CD68) markers. **Results**: All modalities induced an early peak in γH2AX^+^ tumor cells and a pronounced suppression of BrdU^+^ cells, with more sustained effects after isoeffective carbon ions doses, particularly in the HI-tumors. These findings, however, differed strongly between hypoxic and oxic micro-environments. Vascular parameters, diffusion distances, and global and compartment-specific hypoxic fractions showed distinct temporal dynamics between photons and carbon ions in HI-tumors, whereas H-tumors exhibited more moderate and reversible changes. At curative carbon ion doses, there was a late rebound of BrdU-positive tumor cells and increased CD68^+^ macrophage accumulation in chronically hypoxic regions. CD3^+^ T cells showed a biphasic decrease-recovery pattern in HI-tumors largely independent of radiation quality and oxygenation. **Conclusions**: Longitudinal histology revealed modality- and tumor-line-specific trajectories of tumor, vascular, hypoxic, and immune responses after isoeffective photon, proton, and carbon ion irradiations in prostate carcinoma. The more persistent tumor cell damage and distinct vascular response, together with late proliferative and macrophage rebounds under chronic hypoxia after carbon ions, provide mechanistic support for the increased biological effectiveness and highlight hypoxia-driven repopulation and inflammation as key processes.

## Simple Summary

Radiotherapy using protons and carbon ions allows for the delivery of high doses to the tumor while sparing the surrounding normal tissues more effectively compared to conventional photon treatments. In preclinical prostate cancer models, carbon ions have shown more effective and biologically robust tumor control than photons. However, it is still unclear how tumor cells, blood vessels, and immune cells in the tumor change over time after these treatments. In this study, we observed prostate tumors in rats for three weeks after radiotherapy with photons, protons, or carbon ions and analyzed tumor cell damage, proliferation, blood supply, oxygenation, and immune cell infiltration using quantitative fluorescence microscopy. Our findings provide a comprehensive overview of how these tumor and microenvironment features evolve after irradiation with different radiation modalities and dose levels.

## 1. Introduction

Charged particle radiotherapy with protons and carbon ions has emerged as a new modality in radiotherapy to treat tumors at critical locations or tumors that are highly resistant against conventional photon irradiations [1,2]. Both radiation modalities share the physical advantages of a finite range in tissue and an ‘inverted’ depth dose profile, the so-called spread-out Bragg peak (SOBP), allowing for a dose escalation in the tumor while better sparing the surrounding normal tissue [3]. Carbon ions additionally exhibit an increased linear energy transfer (LET), which rises with penetration depth and leads to an increased relative biological effectiveness (RBE), defined as the ratio of absorbed doses for ions and photons that lead to the same biological endpoint [4]. While the RBE of carbon ions typically ranges between 2 and 4 [5], a fixed value of 1.1 is currently adopted for protons [6].

Meanwhile, many different tumor entities are treated with protons or carbon ions [1,2]; however, it is still unknown which tumor patient benefits most from particle irradiations. This holds especially for prostate cancer, which is currently treated either with photons, protons or carbon ions [7]. With respect to this, a better understanding of the radiation response pattern, with regard to radiation type, dose level and the wide range of tumor pathology and micromilieu, could help to assign the optimal treatment to each patient.

Previous studies on three sublines (AT1, HI and H) of the Dunning R3327 rat prostate carcinoma [8] have demonstrated a reduced variability of the carbon ion doses needed to achieve 50% local tumor control (TCD_50_) compared to photon treatments [9]. This indicated that the response to carbon ions is less dependent on the biological tumor characteristics and the presence of resistance factors to photon treatments. This was confirmed in further studies showing that the impact of hypoxia, as one of the most important resistance factors in photon treatments, on local tumor control is considerably smaller for carbon ions than for proton and photon treatments [10,11].

The increased biological effectiveness observed for carbon ions compared to photons is caused by a distinctly different molecular damage pattern to the DNA [12,13]. High-LET particles deposit their energy extremely localized around their tracks, leading to a high density of clustered DNA double strand breaks which are more difficult to repair [14]. Although the increased RBE has been demonstrated in vitro as well as in vivo [4], the underlying mechanisms of how the initial DNA damage develops into clinically relevant endpoints such as local tumor control or normal tissue complications are only poorly understood. However, a better understanding of the development of local tumor control and its determinants is of special importance, and this requires logistically challenging longitudinal studies of radiation response parameters in tumors. While the pattern of DNA damage and the associated repair kinetics following high- and low-LET irradiation have already been studied in detail [15,16], to the authors’ knowledge, no systematic longitudinal histological analysis in tumors considering the impact of radiation modality, dose and hypoxia has been performed yet.

This study presents a comprehensive longitudinal histological analysis in the previously used HI- and H-tumors and quantitatively compares tumor cell-, vascular-, and immune cell-response parameters for oxic and hypoxic tumor regions following isoeffective curative photon and carbon ion irradiations up to 3 weeks after treatment. Additional treatment arms in the HI-tumors include isoeffective curative proton doses as well as isoeffective subcurative photon and carbon ion doses.

## 2. Materials and Methods

### 2.1. Animals, Tumor Sublines, and Tumor Induction

All experiments were conducted in line with German animal welfare regulations and were approved by the local ethics committee in accordance with Directive 2010/63/EU of the European Parliament and Council on the protection of animals used for scientific purposes (animal license number: 35-9185.81/G-197/17 and 35-9185.81/G-151/22, Regierungspräsidium Karlsruhe, Germany). Animals were bred in-house, housed in individually ventilated cages with 3–4 animals per cage and monitored on a daily basis at the DKFZ center of preclinical research.

The Dunning R3327 tumor system has been previously described in detail [8,17,18,19,20], and only a brief summary is given here: The subline R3327-H is a well-differentiated, hormone-sensitive tumor with a low metastatic potential and a volume doubling time of approximately 20 days [17,19,20]. While this subline contains both hormone-sensitive and hormone-insensitive cells, the subline R3327-HI forms a hormone-insensitive tumor characterized by small acini, secretion of epithelial cells, a higher hypoxic fraction and a volume doubling time of approximately 10 days [17,19]. Pathologically, the H-subline corresponds to well-differentiated human prostatic tumors while the HI-subline represents tumor growth following a relapse to hormone therapy [19].

As in previous studies [9,21] tumors were induced by subcutaneous transplantation of tumor fragments into the right hind thigh of male Copenhagen rats (one tumor per animal). Briefly, donor tumors were generated to obtain a sufficient amount of uniform tumor fragments. For this, fresh frozen fragments were thawed, implanted subcutaneously into the anesthetized and analgesia-treated rats, and grown to approximately 20 mm in diameter before resecting and preparing ~2 mm^3^ fragments for transplantation to the adult study animals (weight 335 ± 44 g (1 SD)). Tumors that did not grow or reach the required size were excluded and replaced.

### 2.2. Irradiation Setup

Irradiations essentially followed previously established protocols and are only briefly described here [9,21]. Tumors were irradiated at a mean tumor diameter of 10 mm using a single beam from the rear direction. During irradiation, rats were fixed in a customized bed positioned by means of room lasers. Rats were anesthetized by breathing 100% oxygen (Guttroff GmbH, Wertheim, Germany) with a mixture of 2.5 Vol% of isoflurane (photons, CP-Pharma Handelsgesellschaft mbH, Burgdorf, Germany) or sevoflurane (protons and carbon ions, Zoetis, Parsippany, NJ, USA) starting 10 min prior and continuing during irradiation. The rats’ eyes were covered with Bepanthen^®^ (Bayer Vital GmbH, Leverkusen-Wiesdorf, Germany) to prevent drying.

Photon irradiations were performed in a clinical 6 MV linear accelerator (Ethos, Varian Medical Systems, Palo Alto, CA, USA) at the DKFZ using a horizontal beam with a 20 × 20 mm^2^ field size (90% isodose diameter: 16 mm, dose rate 600 monitor units/min). As in previous studies [9,21], the tumor was irradiated using a 10 mm polymethylmethacrylate (PMMA) bolus directly in front and a second 15 mm PMMA bolus behind the tumor. Prior to irradiation, the tumor position was checked using low-dose cone beam computed tomography (CBCT).

As in previous studies [9,11,21], proton and carbon ion irradiations were performed at the experimental beamline of the Heidelberg Ion Beam Therapy Center (HIT) using a horizontal beam with a field size of 18 × 18 mm^2^ (90% isodose: 16.5 × 16.5 mm^2^), delivered by active pencil beam scanning [22,23]. The tumor was located at the center of a 20 mm spread-out Bragg peak (SOBP) by placing a 39.9 mm PMMA bolus directly in front and a 15 mm PMMA bolus behind the tumor. As a result, the first 5 mm of the SOBP on the central axis were located in the proximal bolus while the last 5 mm were located in the distal bolus.

### 2.3. Design of Histological Study

**Figure 1:**
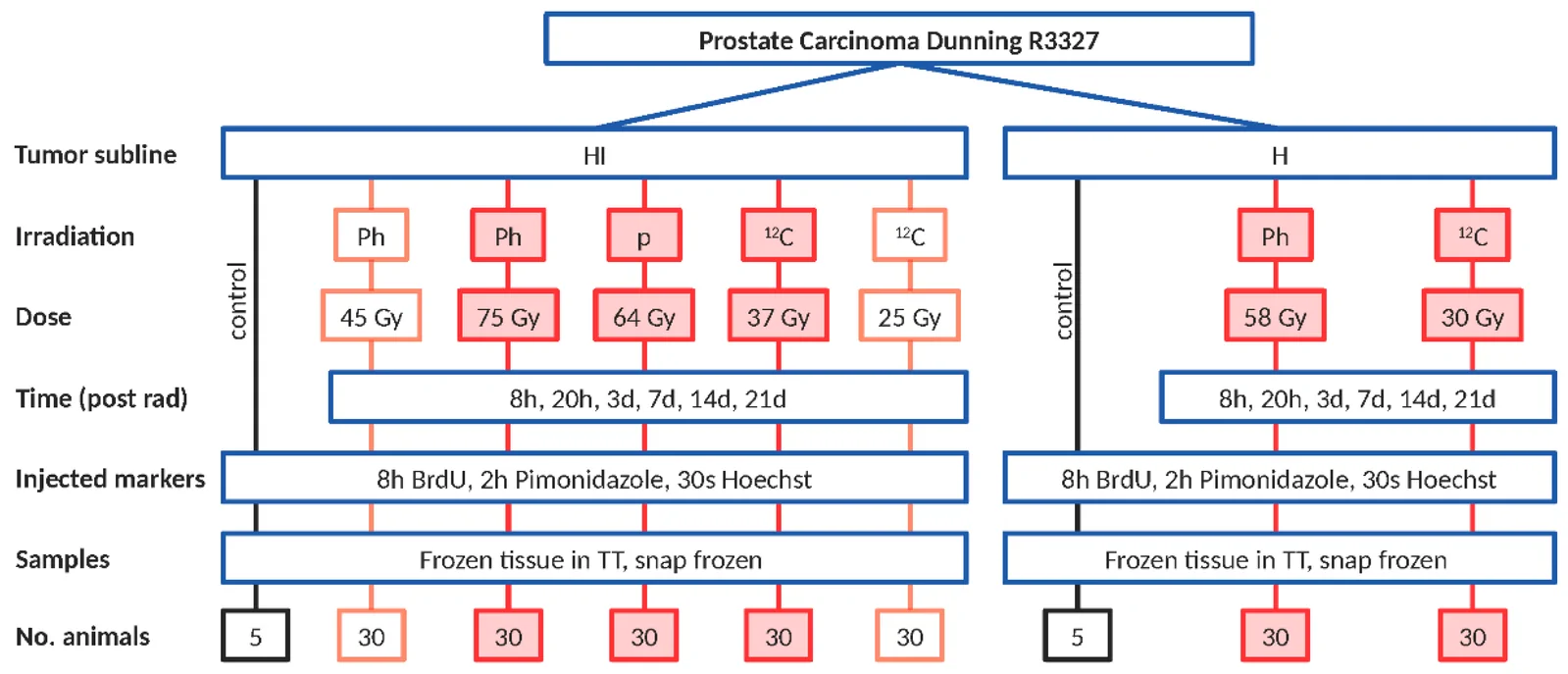
Experimental design. Two sublines (HI, H) of the prostate carcinoma Dunning R3327 were irradiated either with photons (Ph), protons (p) or carbon ions (^12^C) using either isoeffective curative (red filled boxes) or isoeffective subcurative (red open boxes) doses (experimental arms). Unirradiated animals served as controls (black open boxes). After marker injection, tumors were dissected prior or at six time points after irradiation. Tumor samples were snap frozen and embedded in TissueTek^®^ (TT) for further analysis. A total of 5 tumor samples per experimental arm and time point were available for histological analysis.

Figure 1 displays the design of the histological study in the HI- and H-tumors including the employed radiation modalities and dose levels as experimental arms. After marker injection (see Section 2.4), irradiated tumors were dissected either at 8 h, 20 h, 3 d, 7 d, 14 d or 21 d after irradiation (see Section 2.4). In addition, unirradiated tumors served as controls. A total of 5 tumor samples per experimental arm and time point were used for histological analysis to account for the heterogeneity of the tumors, except for curative protons at 20 h, where we had one dropout animal.

Dose levels for the three radiation modalities were selected, either isoeffective curative or isoeffective subcurative (HI-tumor only). A curative dose implies a tumor control probability (TCP) of 100%, while a subcurative dose leads to TCP of 0%. The attribute “isoeffective” indicates that the dose levels for the different radiation modalities considered the RBE to arrive at the same endpoint (100% or 0% TCP). The relation between dose and TCP for the three radiation modalities was known from previous dose–response studies [9,11]. For the HI-tumor, the isoeffective curative doses were 75 Gy (photons), 64 Gy (protons) and 37 Gy (carbon ions) while the isoeffective subcurative dose were 45 Gy (photons) and 25 Gy (carbon ions). For the H-tumor, only isoeffective curative doses were applied: 58 Gy for photons and 30 Gy for carbon ions.

A strict randomization of the animals to the different treatment arms and groups was not possible due to logistic constraints (two irradiation devices, beam time limitations, tumor growth times, animal breeding) and therefore the experiments had to be executed consecutively. However, it was ensured that each treatment group was irradiated at least during two beam times and the time points of histological evaluation were fixed at the time of irradiation. Quality check of fluorescence images was unblinded; however, the analysis itself was automized by scripts and therefore quasi-blinded.

### 2.4. Tumor Dissection

Similar to a previous study [10], the following markers were injected intraperitoneally (i.p.) prior to tumor dissection. Rats received bromodeoxyuridine (BrdU, 100 mg/kg, dissolved in 0.9% NaCl; Sigma-Aldrich Chemie GmbH, Taufkirchen, Germany) 8 h before tumor dissection to label proliferating cells. Pimonidazole (60 mg/kg, dissolved in 0.9% NaCl, Hypoxyprobe, Inc., Burlington, MA, USA) was administered 2 h prior to tumor dissection to mark hypoxia. Under isoflurane anesthesia, animals were prepared surgically, and 30 s before sacrifice by cardiac puncture, the perfusion marker Hoechst 33,342 (15 mg/kg, dissolved in 0.9% NaCl, Sigma-Aldrich Chemie GmbH, Taufkirchen, Germany) was injected into the left ventricle to visualize perfused tumor regions. Dissected tumors were rinsed in PBS, bisected, and either embedded in TissueTek^®^ and frozen on dry ice or snap-frozen in liquid nitrogen. Samples were stored at −80 °C until further processing.

### 2.5. Immunohistochemistry

Tumors were cryosectioned (7 µm, three sections per tumor, 150 µm apart) using a Microm™ HM 560 Cryostat (Thermo Fisher Scientific, Dreieich, Germany). Sections were fixed in methanol (1 min, 4 °C) and acetone (2 min, 4 °C) and stored at −20 °C.

For protein detection, cryosections were thawed, and antigen retrieval (10 mM sodium-citrate buffer, pH 6.0, 95 °C, 30 min) was performed when required. Sections were blocked with DAKO protein block or 5% BSA and incubated with primary antibodies overnight at 4 °C, followed by secondary antibodies incubation for 1 h at room temperature. Nuclei were counterstained with DAPI (100 ng/mL, 3 min, Carl Roth GmbH + Co. KG, Karlsruhe, Germany).

Triple staining of BrdU (#11170376001, RocheMannheim, Germany), γH2AX (#2577, 1:1000, Cell Signaling), and pimonidazole (FITC-conjugated, clone 4.3.11.3, 1:100, Natural Pharmacia International Inc, Burlington, MA, USA) was used to analyze proliferation, DNA damage, and hypoxia, respectively. Immune cell infiltration was assessed using antibodies against CD68 (macrophages, #MCA341R, 1:1000, Bio-Rad Antibodies) and CD3 (T cells, #NB 600-1441, 1:100, Novus Biologicals, Centennial, CO, USA), combined with pimonidazole to differentiate oxic and hypoxic areas. Vascular structures and perfusion were visualized using CD31 (#AF3628, 1:800, Bio-Techne GmbH, Düsseldorf, Germany) and Hoechst 33,342, together with pimonidazole to evaluate hypoxia and metabolic adaptation.

### 2.6. Image Processing

Fluorescence images were acquired using an Axio Scan. Z1 microscope (Carl Zeiss Microscopy, Jena, Germany) with 20× magnification. Automated focus mapping was performed using the DAPI channel, and images were stitched and saved in CZI format.

#### 2.6.1. Image Analysis

Image data were organized using the OMERO v5.6.9 platform (Open Microscopy Environment, DKFZ servers) and analyzed with QuPath v0.5.0. Prior to analysis, images were preprocessed to ensure high-quality input data. Vital tumor tissue was identified using a pixel classifier (intensity threshold 1000–1200, 5.2 μm/px resolution) on the DAPI or Hoechst channel, applying a Gaussian prefilter (σ = 1) to exclude necrotic areas. Objects smaller than 100,000 μm^2^ or with holes below 10,000 μ^2^ were removed. Channels were renamed according to the corresponding fluorophores, and artifacts such as debris, overlapping tissue, or air bubbles were manually excluded. Image and staining quality were rated on a five-point scale within OMERO. Cell detection was performed on the DAPI or Hoechst channel at 0.5 μm pixel size to balance accuracy and processing time. Nuclei were identified using QuPath with a background radius of 10 μm, sigma 2, minimum and maximum areas of 20^2^ m^2^ and 400^2^ μm^2^, respectively, and a threshold of 200. Nuclei were split by shape, expanded by 4 μm to define cell boundaries, and measurements were extracted for each detected cell.

Machine learning classifiers were trained with five training images per channel. Training images were annotated to train random trees (RTrees) classifiers for cell detection and classification, including BrdU, γH2AX, and cytoplasmic markers. Vessel structures were classified on the CD31 channel, distinguishing perfused (CD31^+^/Hoechst^+^) from non-perfused (CD31^+^/Hoechst^−^) vessels.

Pimonidazole^+^/Hoechst^+^ cells were classified as cyclic hypoxic, while Pimonidazole^+^/Hoechst^−^ cells were categorized as diffusion- or perfusion-limited based on proximity to perfused or non-perfused vessels. Diffusion distance was quantified as the mean distance of 95% of the cells to the nearest perfused vessels.

#### 2.6.2. Batch Processing and Quantification

Classifiers were trained separately for each staining batch. Images were batch-processed, and cell-level data was exported to CSV files. Marker-positive and -negative cells were quantified per tumor and treatment condition, with hypoxic/oxic classification based on pimonidazole. Poor-quality images (OMERO rating ≤ 2) were excluded. Data visualization was performed using the packages *dplyr*, *tidyr*, and *ggplot2* of RStudio v2024.09.1.

### 2.7. Statistics

Statistical analyses were performed in RStudio (v2024.09.1) using the *carData* and *multcomp *packages of RStudio v2024.09.1. Group comparisons were conducted by one-way ANOVA with Dunnett’s post hoc test versus controls, and differences between radiation modalities at each time point were assessed by ANOVA with Tukey HSD post hoc test. *p* ≤ 0.05 was considered statistically significant.

## 3. Results

### 3.1. Tumor Cell Response

**Figure 2:**
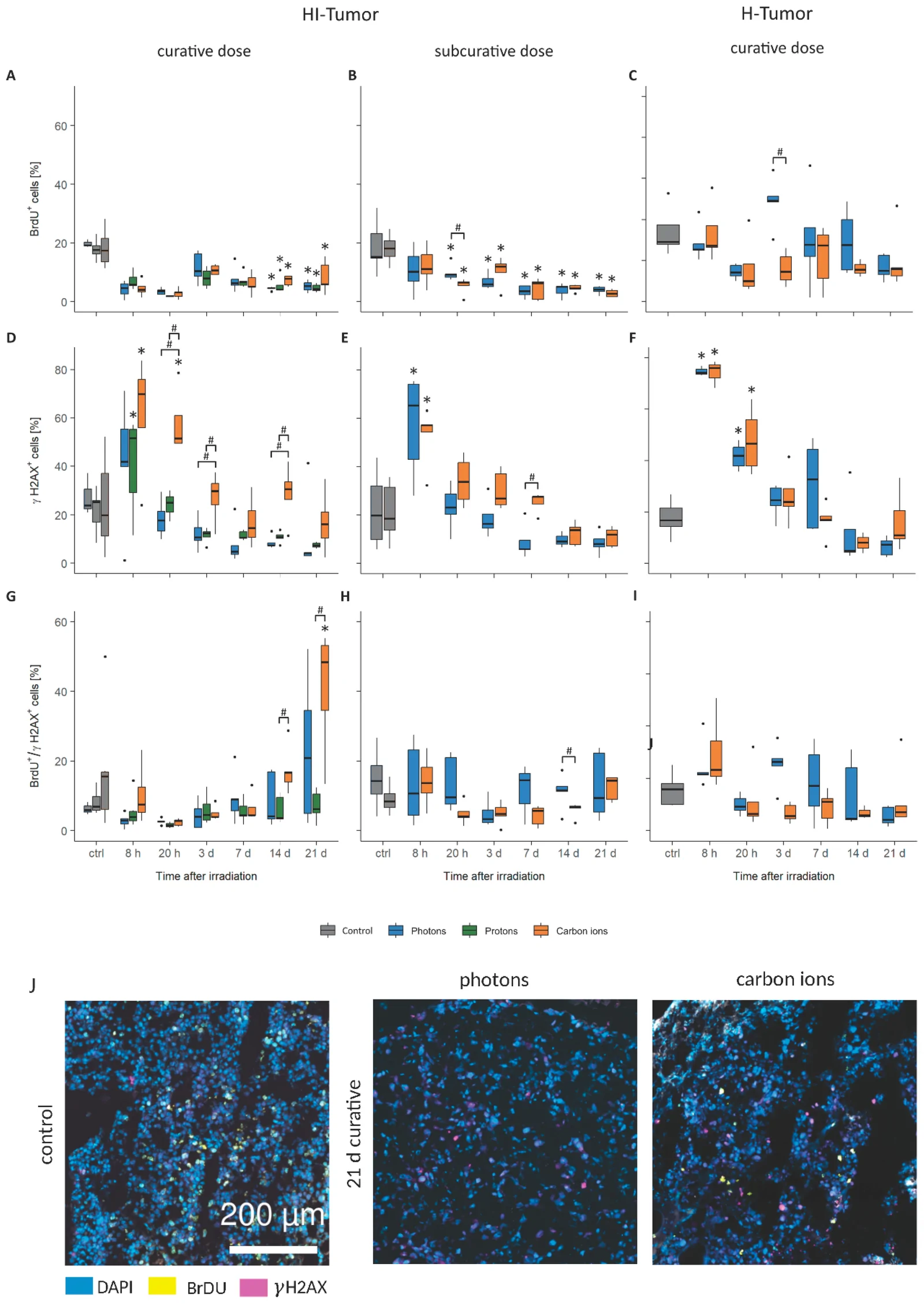
(**A**–**I**). All BrdU^+^ (**top**), γH2AX^+^ (**middle**) and BrdU^+^/γH2AX^+^ (**bottom**) cells in the HI-tumors ((**left**) and (**middle**)) and H-tumors (**right**) for different beam modalities and for curative ((**left)** and (**right**)) and subcurative ((**middle**), HI-tumor only) doses. Samples were evaluated prior to and at six time points after irradiation. * and # indicate significant differences with respect to controls or between radiation modalities, respectively (*p* < 0.05). Separations of these quantities according to oxic and hypoxic regions are shown in Figures S1 and S2**,** respectively. (**J**). Representative microscopic fluorescence images of the HI-tumor showing BrdU^+^/γH2AX^+^ at 21 d after irradiation with curative photon and carbon ion doses in comparison to a control. (Boxplots–median, IQR, whiskers: 1.5 IQR, dots: outliers).

To analyze proliferation and DNA damage in the HI- and H-tumors after irradiation, the fluorescence staining of all BrdU^+^, γH2AX^+^ and BrdU^+^/γH2AX^+^ cells at different time points were compared for different beam modalities, separated according to curative and subcurative (only HI-tumor) doses (Figure 2). In HI-tumors, BrdU^+^ and BrdU^+^/γH2AX^+^ cells generally decreased significantly after irradiation, independent of radiation modality and dose level. In contrast, γH2AX^+^ cells showed a strong initial increase and a subsequent decrease below control level for both curative and subcurative doses, with significantly higher values and a delayed decrease for carbon ions. For curative doses, this delay was more pronounced, and no difference was found between photons and protons. For the H-tumor, a similar trend was found after curative photon and carbon ion doses, however without a delayed decrease in γH2AX^+^ cells.

**Figure 3:**
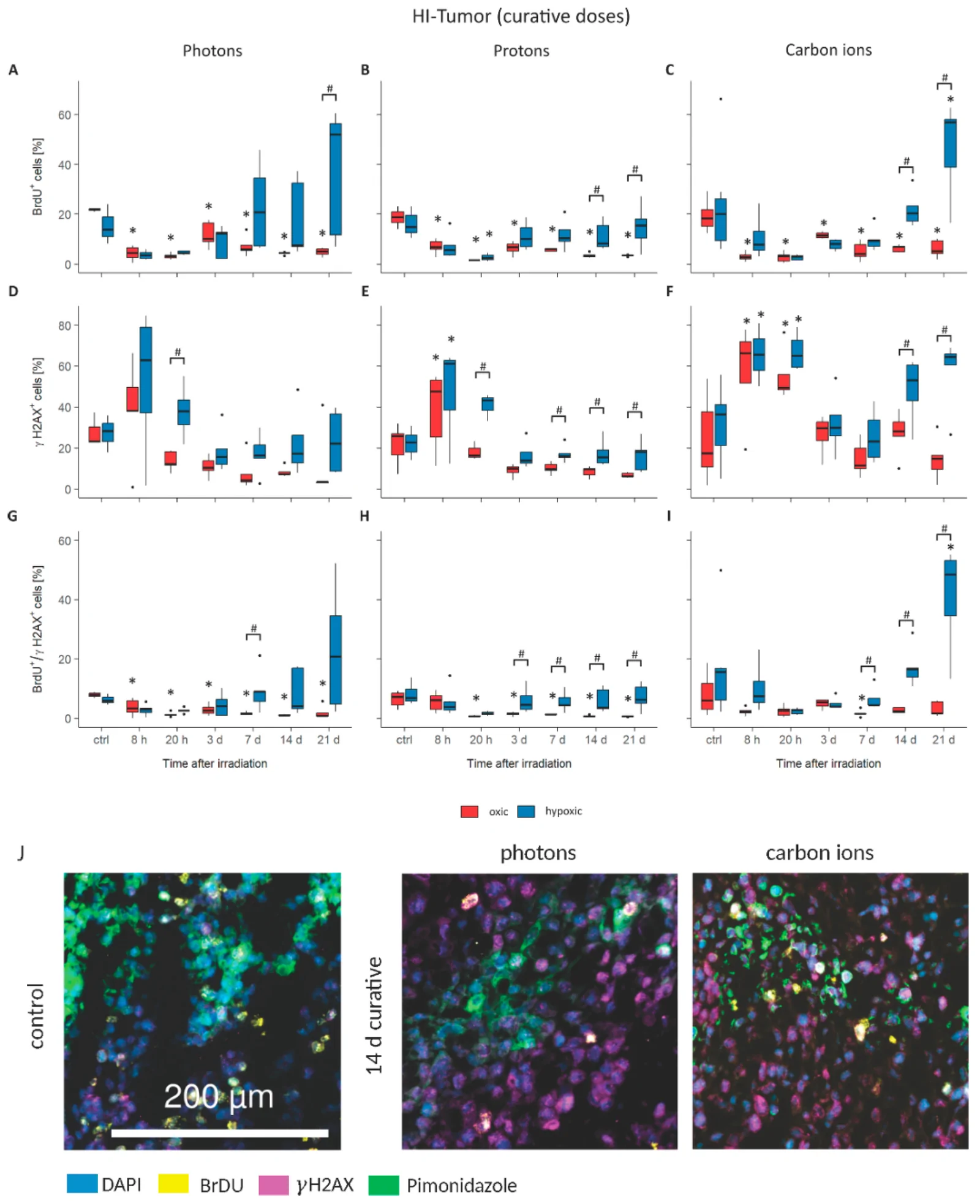
(**A**–**I**). Comparison of BrdU^+^ (**top**), γH2AX^+^ (**middle**) and BrdU^+^/γH2AX^+^ (**bottom**) cells in oxic (Pimonidazole^−^) and hypoxic (Pimonidazole^+^) regions in the HI-tumor for curative doses of photons (**left**), protons (**middle**) or carbon ions (**right**). Samples were evaluated prior to and at six time points after irradiation. * and # indicate significant differences with respect to controls or between oxic and hypoxic regions, respectively (*p* < 0.05). (**J**). Representative immune fluorescence stainings of BrdU^+^/γH2AX^+^ in hypoxic and normoxic areas of the tumor before and 14 d after curative irradiation with either photons or carbon ions. (Boxplots–median, IQR, whiskers: 1.5 IQR, dots: outliers).

**Figure 4:**
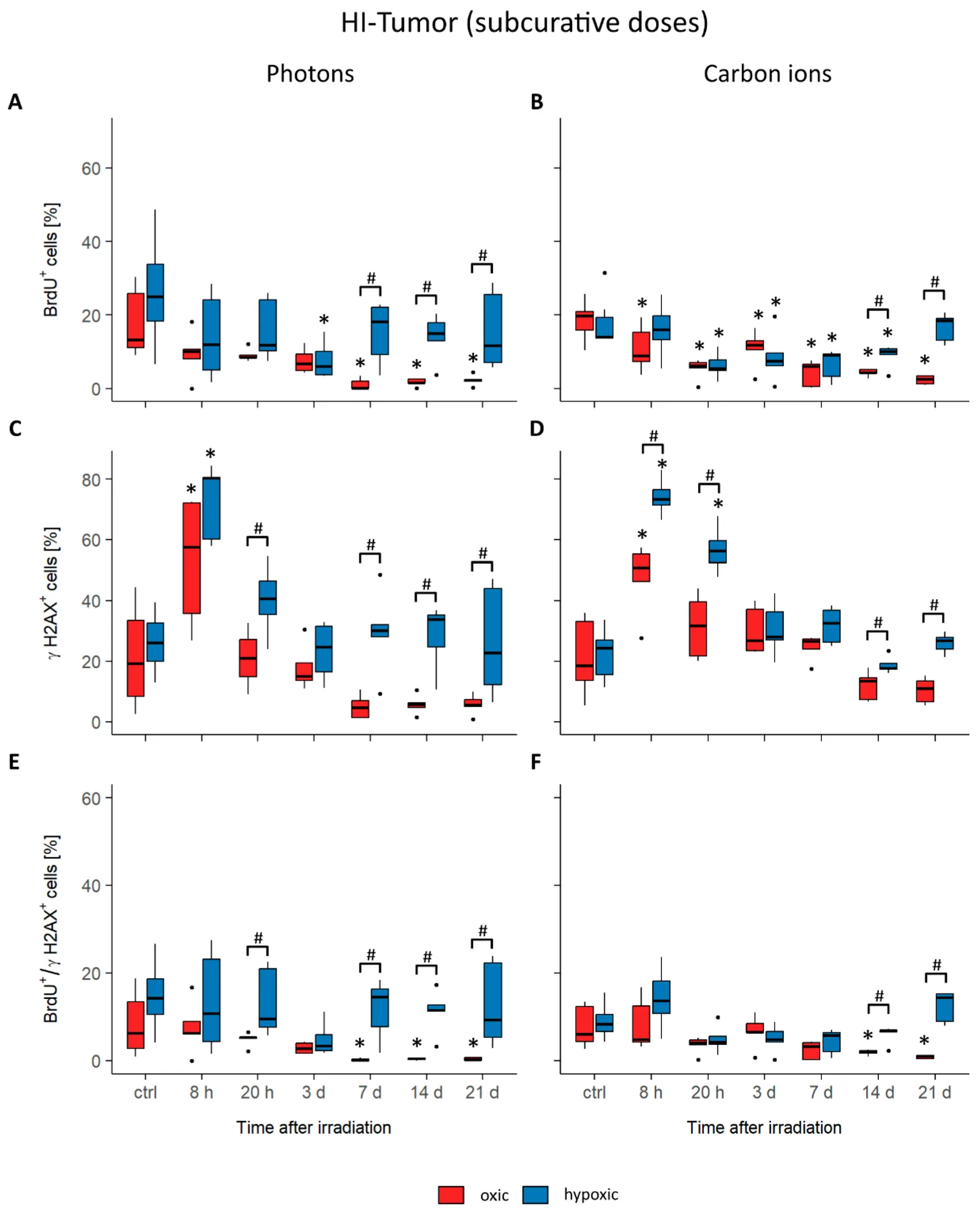
(**A**–**F**) Comparison of BrdU^+^ (**top**), γH2AX^+^ (**middle**) and BrdU^+^/γH2AX^+^ (**bottom**) cells in oxic (Pimonidazole^−^) and hypoxic (Pimonidazole^+^) regions in the HI-tumor after subcurative doses of photons (**left**) or carbon ions (**right**). Samples were evaluated prior to and at six time points after irradiation. * and # indicate significant differences with respect to controls or between oxic and hypoxic regions, respectively (*p* < 0.05). (Boxplots–median, IQR, whiskers: 1.5 IQR, dots: outliers).

Comparing oxic (Pimonidazole^−^) and hypoxic (Pimonidazole^+^) regions after curative doses in HI-tumors demonstrated that after the significant initial decrease, BrdU^+^ and BrdU^+^/γH2AX^+^ cells rose strongly in hypoxic regions while they remained at low levels in areas with oxic cells (Figure 3). γH2AX^+^ cells exhibited an initial increase, followed by a decrease below control level followed by another increase for hypoxic but not for oxic cells. The decrease was earlier and stronger for oxic than for hypoxic cells. For subcurative photon and carbon ion doses, BrdU^+^ and BrdU^+^/γH2AX^+^ cells also decreased below control level under oxic, but remained essentially unchanged in hypoxic regions (Figure 4). After subcurative doses, γH2AX^+^ cells behaved similarly as after curative doses.

Figures S1 and S2 compare the BrdU^+^, γH2AX^+^ and BrdU^+^/γH2AX^+^ cells in the HI-tumor for the different radiation modalities separately in oxic and hypoxic regions, respectively. In oxic regions, only a few significant but small differences were found between radiation modalities for BrdU^+^ and BrdU^+^/γH2AX^+^ cells after curative and subcurative doses. Only for γH2AX^+^ cells, a clear significant difference between radiation modalities was seen, originating from the delayed decrease for carbon ions, which was more pronounced for curative doses. Under hypoxia, no significant differences between radiation modalities were found, except for a stronger increase in BrdU^+^/γH2AX^+^ cells at 14 d and 21 d after curative carbon ion doses. In addition, a trend for a delayed decrease in γH2AX^+^ cells for carbon ions after curative and subcurative doses was seen.

**Figure 5:**
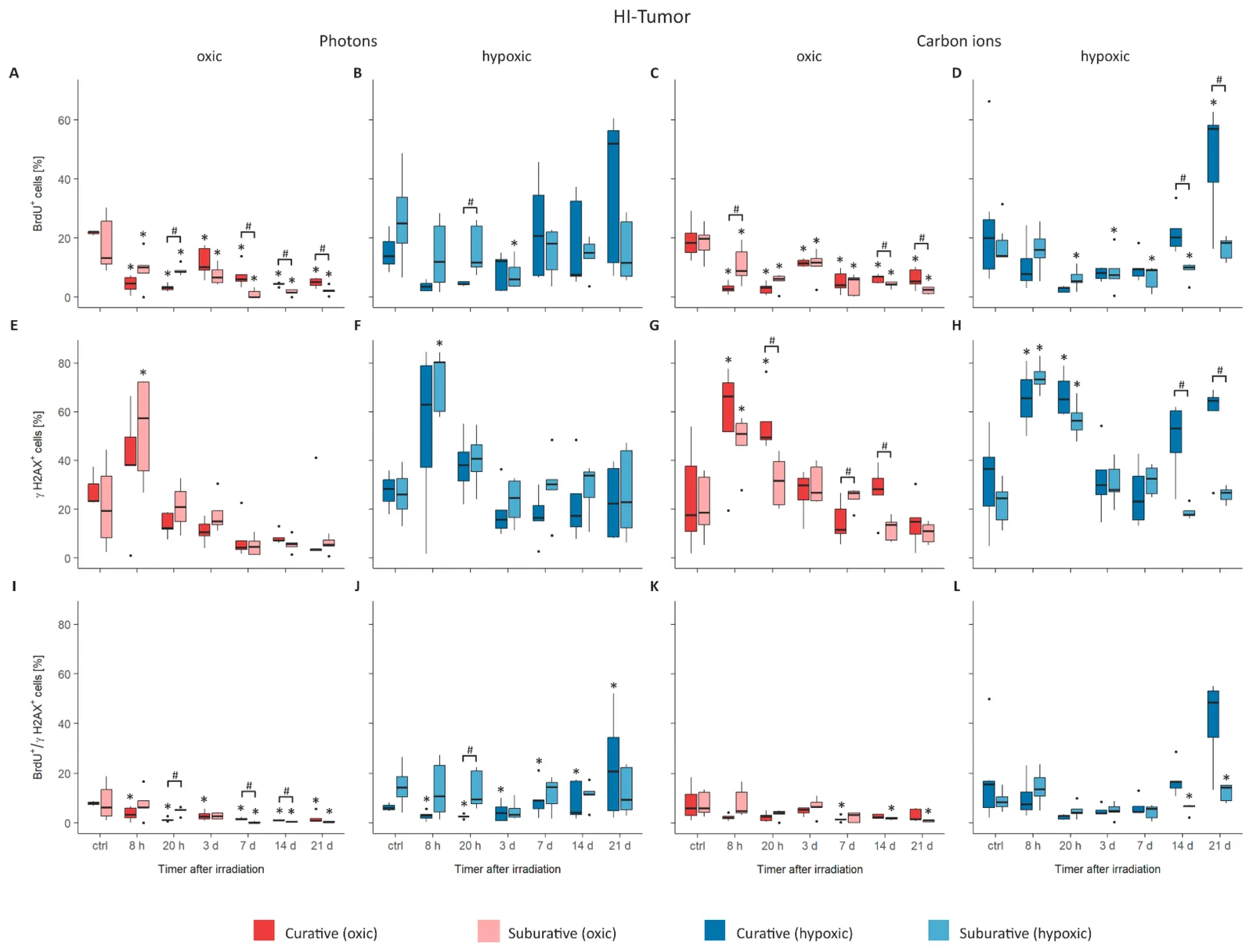
(**A**–**L**) Comparison of BrdU^+^ (**top**), γH2AX^+^ (**middle**) and BrdU^+^/γH2AX^+^ (**bottom**) for curative and subcurative doses of photons (**left**) or carbon ions (**right**), both separated according to oxic and hypoxic regions in the HI-tumor. Samples were evaluated prior to and at six time points after irradiation. * and # indicate significant differences with respect to controls or between dose levels, respectively (*p* < 0.05). (Boxplots–median, IQR, whiskers: 1.5 IQR, dots: outliers).

To visualize the impact of dose level, Figure 5 directly compares the changes in BrdU^+^, γH2AX^+^ and BrdU^+^/γH2AX^+^ cells in the HI-tumor for curative and subcurative photon and carbon ion doses, separated according to oxic and hypoxic regions. The general trend for curative and subcurative doses was the same for both radiation modalities and oxygen regions, however with a much stronger increase for curative carbon ion doses in hypoxic regions at 14 and 21 days. Although some differences between curative and subcurative doses in oxic regions were significant, most of them were rather small.

### 3.2. Vascular Response

**Figure 6:**
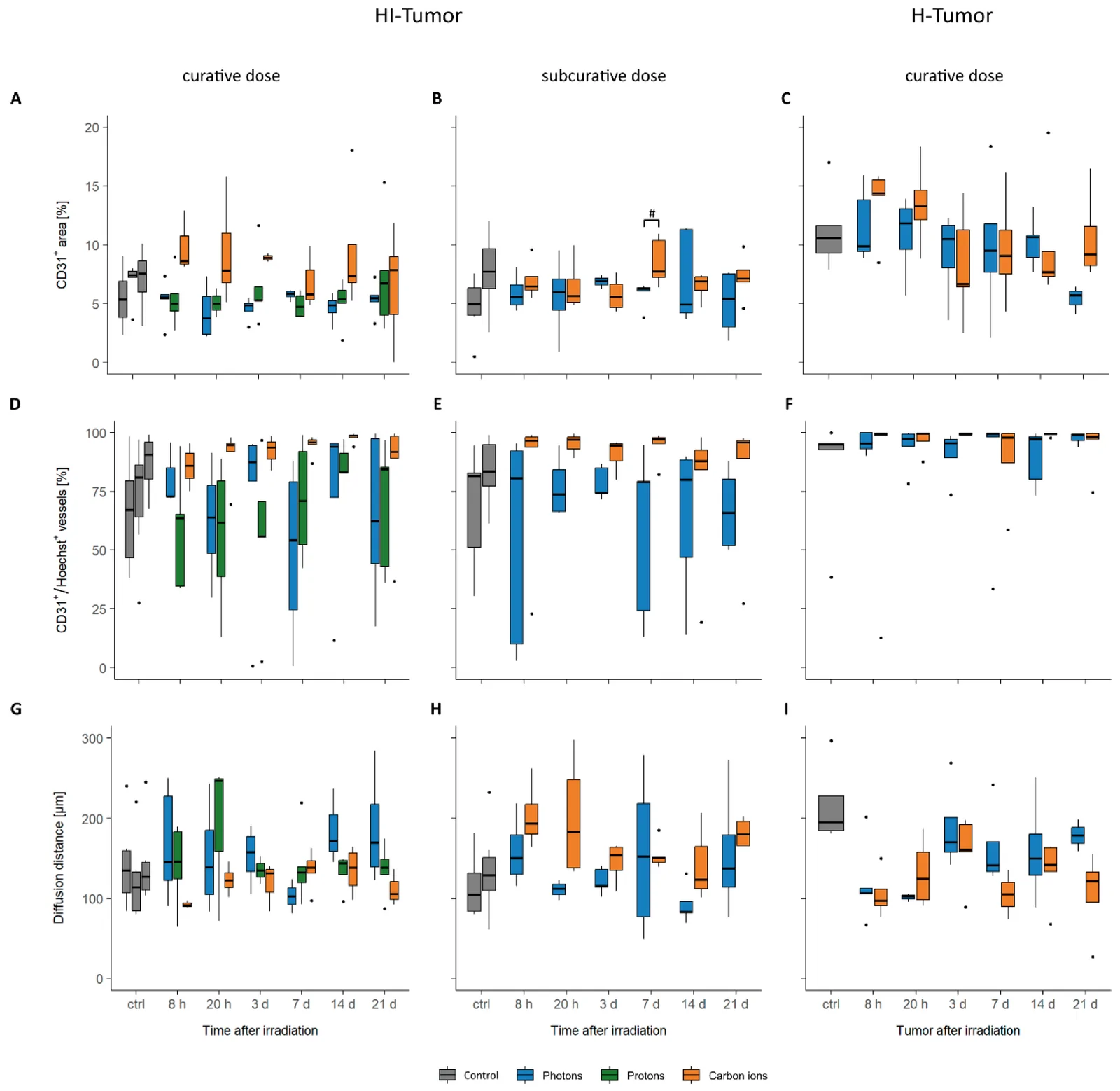
(**A**–**I**) CD31^+^ (**top**), CD31^+^/Hoechst^+^ (**middle**) and diffusion distance (**bottom**) for curative (**left** and **right**) and subcurative (**middle**, HI-tumor only) irradiated with different radiation modalities. Samples were evaluated prior to and at six time points after irradiation. * and # indicate significant differences with respect to controls or between radiation modalities, respectively (*p* < 0.05). (Boxplots–median, IQR, whiskers: 1.5 IQR, dots: outliers).

**Figure 7:**
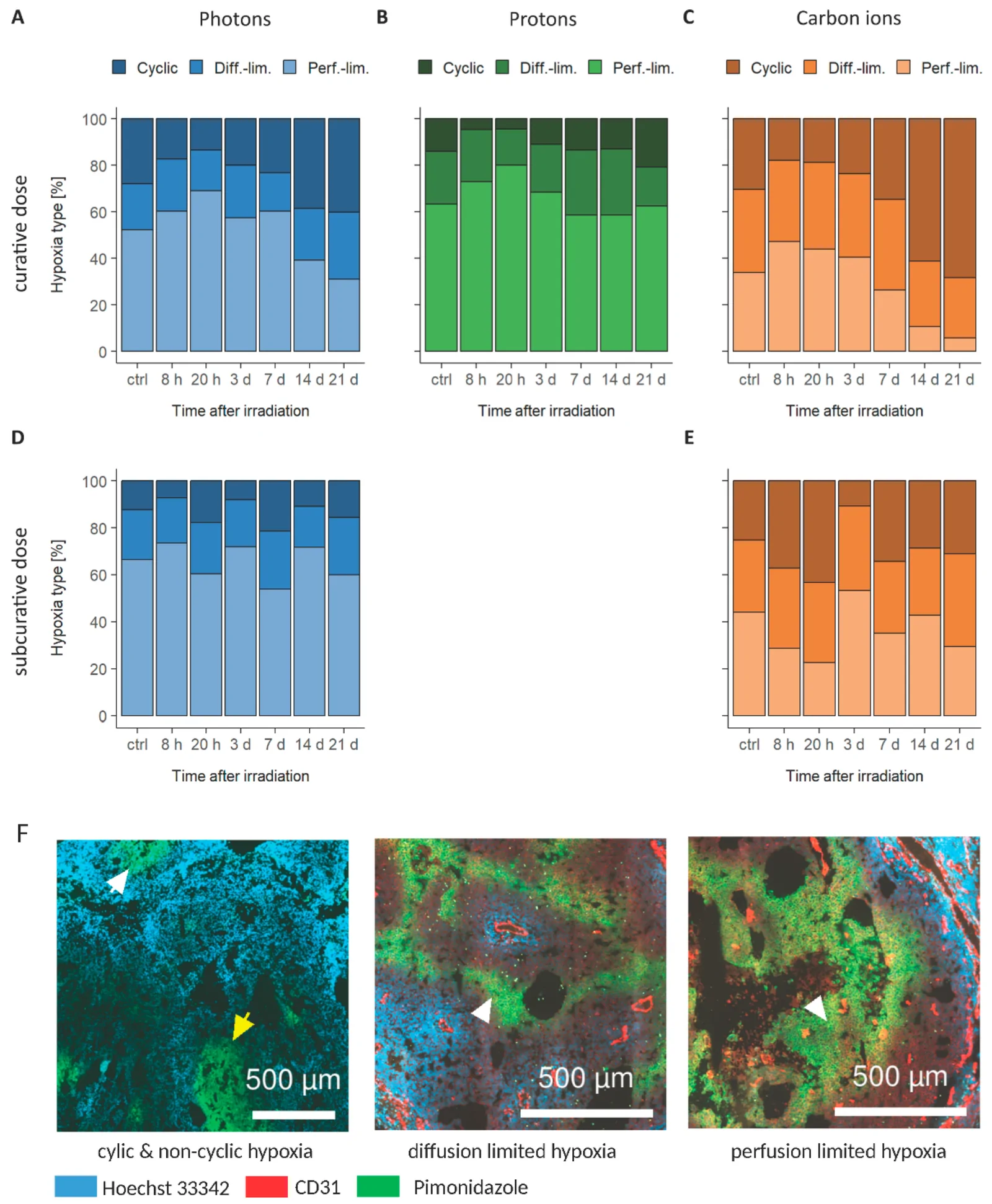
(**A**–**E**). Classification of hypoxic cells in the HI-tumor into cyclic, diffusion-limited and perfusion-limited hypoxia, based on the perfusion status (Hoechst^+/−^) of each cell and that of the nearest vessel after delivering curative or subcurative doses with different radiation modalities. (**F**). Representative immunofluorescent stainings distinguishing cyclic (white arrow) versus non-cyclic hypoxia (yellow arrow, (**left image**)), diffusion limited hypoxia ((**middle image**), arrow), and perfusion limited hypoxia ((**right image**), arrow).

To investigate the vascular response in the HI- and H-tumor after irradiation, the CD31^+^, CD31^+^/Hoechst^+^ areas and the related diffusion distances at different time points were compared for different beam modalities, separated according to curative and subcurative (HI-tumor only) doses (Figure 6). No clearly significant differences were found, although there was a trend for slightly more CD31^+^and CD31^+^/Hoechst^+^ cells in the HI-tumor after carbon ions as compared to photons and protons. Classification of hypoxia types revealed an increase in cyclic and diffusion-limited, relative to perfusion-limited, hypoxia after curative and subcurative doses of carbon ions in comparison to photons and protons (curative doses only) (Figure 7).

### 3.3. Immune Cell Response

**Figure 8:**
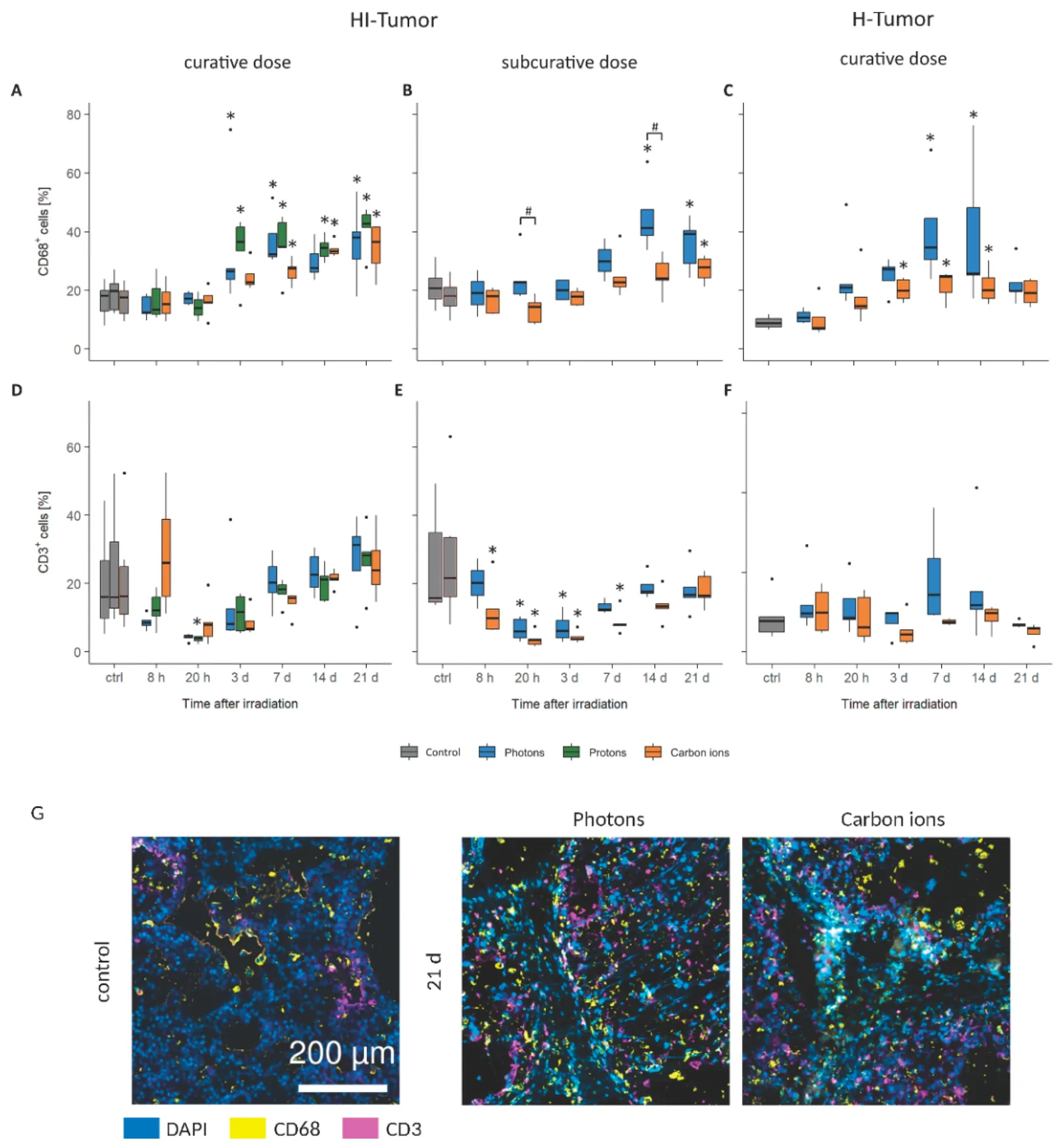
(**A**–**F**). All CD68^+^ (**top**) and CD3^+^ (**bottom**) cells in the HI-tumor (**left** and **middle**) and H-tumor (**right**) for different beam modalities and for curative (**left** and **right**) and subcurative (**middle**, HI-tumor only) doses. Samples were evaluated prior to and at six time points after irradiation. * and # indicate significant differences with respect to controls or between radiation modalities, respectively (*p* < 0.05). Separation of these quantities according to oxic and hypoxic regions are shown in Figures S5 and S6. (**G**). Representative immune fluorescence stainings 21 d after irradiation with photons or carbon ions. (Boxplots–median, IQR, whiskers: 1.5 IQR, dots: outliers).

**Figure 9:**
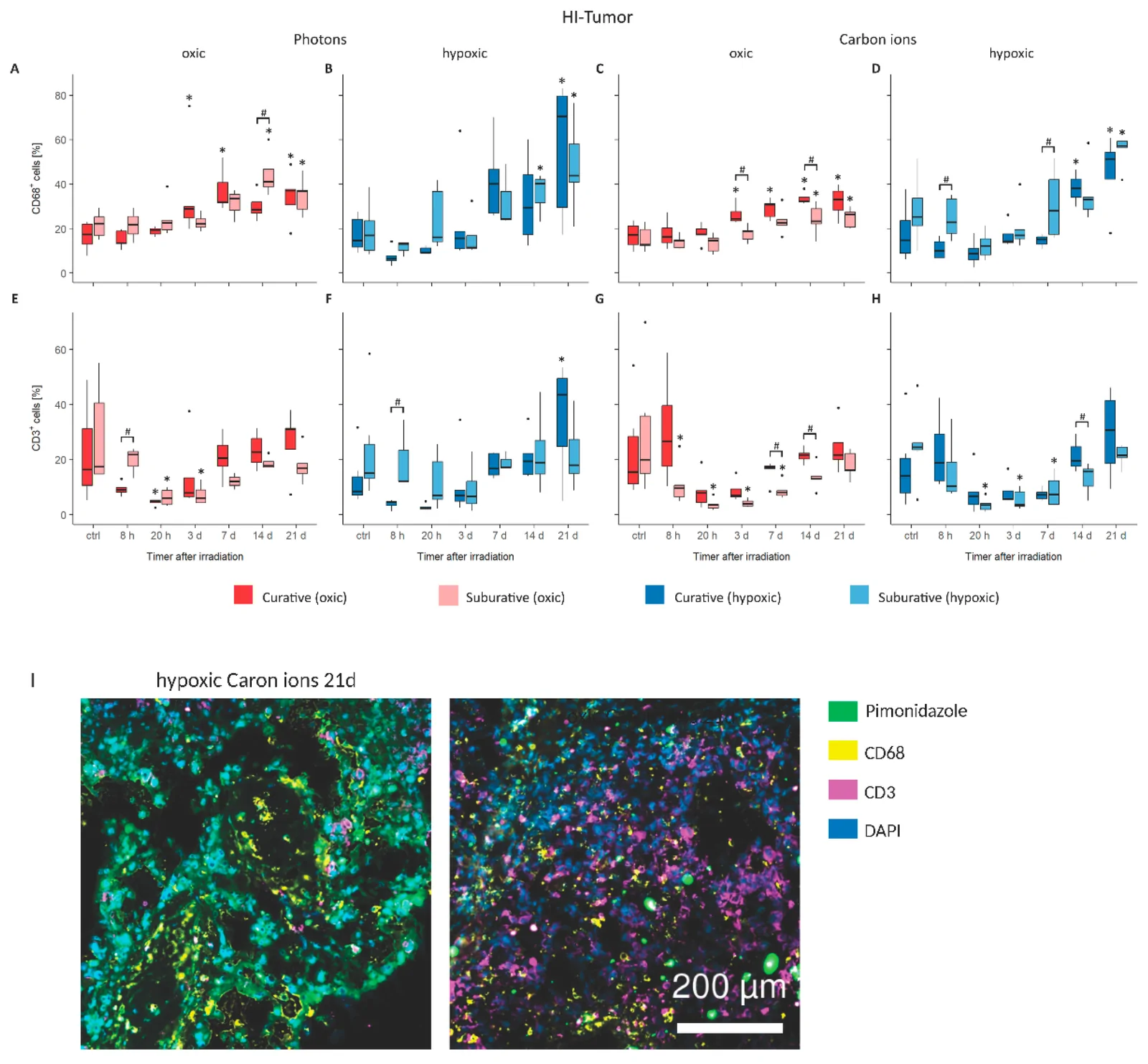
(**A**–**H**). Comparison of CD68^+^ (**top**) and CD3^+^ (**bottom**) for curative and subcurative as well as for oxic and hypoxic regions in the HI-tumor irradiated with photons (**left**) or carbon ions (**right**). Samples were evaluated prior to and at six time points after irradiation. * and # indicate significant differences with respect to controls or between dose level, respectively (*p* < 0.05). (**I**). Representative image of the same HI-tumor slide comparing hypoxic versus oxic areas. (Boxplots–median, IQR, whiskers: 1.5 IQR, dots: outliers).

To analyze the immune response in the HI- and H-tumor after irradiation, the fluorescence staining of all CD68^+^ and CD3^+^ cells at different time points were compared for different beam modalities, separated according to curative and subcurative (HI-tumor only) doses (Figure 8). There was a significant increase in CD68^+^ cells with time for all radiation modalities, for both dose levels and both tumors. This increase, however, was less for carbon ions than for photons and protons. Figure S3 shows further that the increase was delayed in hypoxic relative to oxic regions for curative doses of all radiation modalities; however, at 21 d, the highest value was found in hypoxic regions. For subcurative doses, no clear difference between oxic and hypoxic cells was found (Figure S4). As indicated in Figure 9, the amount of CD68^+^ cells in the HI-tumor was slightly, but systematically, lower for subcurative compared to curative carbon ion doses in oxic regions, but no clear difference was found for photons (oxic and hypoxic regions) or carbon ions in hypoxic regions (Figure 9). Comparing CD68^+^ cells for different radiation modalities separately for oxic and hypoxic regions revealed a delayed increase for carbon ions in oxic (Figure S5) but not in hypoxic (Figure S6) regions.

After irradiation of the HI-tumor with curative and subcurative doses, the amount of all CD3^+^ cells first decreased and then increased again, independent of radiation type, while no clear tendency was found in the H-tumor (Figure 8). Essentially the same behavior was found for the HI-tumor when separating the CD3^+^ cells according to oxic and hypoxic regions (Figures S3–S6). As indicated in Figure 9, the increase in CD3^+^ cells in oxic regions was slightly larger after curative than after subcurative doses of photons and carbon ions, while no clear difference was found in hypoxic regions.

## 4. Discussion

Several studies investigated the effectiveness of ion beams relative to photons and a clear impact of LET was found [4,24,25,26]. Clinically, the increased effectiveness is considered by the RBE, which directly enters dose prescriptions [4,5]. While it has been shown that the DNA damage pattern is distinctly different for low- and high-LET radiation [12,13], the potentially different mechanism leading to the clinically relevant endpoint local tumor control remained unclear and, to the authors’ best knowledge, this is the first systematic study that investigates histological parameters for different tumor types, radiation modalities, and dose and oxygen levels over time after irradiation.

It appears quite evident that the radiation response of tumors is initiated by DNA damage and that this parameter together with tumor cell proliferation (measured by γH2AX and BrdU) is decisive for local tumor control. Investigating this in vivo is crucial because changes in the tumor microenvironment and activation or suppression of the immune system can profoundly influence radiation effects by inhibiting or promoting tumor growth. In addition, hypoxia (assessed by Pimonidazole) as the most important resistant factor plays an important role [27]. With respect to the HI-tumor cell response, the two key findings in response to the radiation quality were: (i) after irradiation, DNA damage increased significantly before it decreased again, and the increase was significantly higher and the decrease was delayed for carbon ions relative to photons and proton; (ii) irradiation-induced proliferation inhibition was independent of radiation modality and dose level. As the DNA is the primary target, the increase in DNA damage was expected and the delayed decrease for high-LET radiation has been observed previously by Schmid et al. [15] and may be explained by the longer time needed to repair complex DNA-cluster damage. However, it has to be noted that γH2AX is known to increase already after 10–60 min [28], so the maximum γH2AX signal was likely not captured in our study. In our study, γH2AX was assessed at 8 h, 20 h, 3 d, 7 d, 14 d, and 21 d after radiotherapy, i.e., at time points when the initial γH2AX peak has subsided and the remaining signal is generally considered to reflect residual, more slowly repaired DNA damage. While the inhibition of cell proliferation following radiation exposure, resulting from DNA damage-induced cell cycle arrest, growth suppression, and eventual cell death is well established [29], the lack of differences observed between photon, protons and carbon ions may be explained by the use of isoeffective dose levels, which were either curative of subcurative. This implies that the RBE determined for the endpoint local tumor control is also valid for this histological endpoint. Since effects on proliferation behaved similarly for all γH2AX^+^ cells, it appears likely that DNA damage is responsible for the observed changes in proliferation. Although proliferation was not radiation modality-dependent, different kinetics in the DNA damage pattern were observed, indicating mechanistic differences in the response to low- and high-LET radiation.

As a further important result, we found that BrdU incorporation post irradiation was suppressed more for oxic than for hypoxic cells. BrdU labels proliferating, or, more generally, DNA-synthesizing cells, and after irradiation, BrdU incorporation can additionally reflect repair-associated DNA synthesis. While BrdU incorporation of oxic cells remained at a low level, we found that DNA-synthesis in hypoxic cells increased strongly for both dose levels at the end of the observation period. This finding may be explained by the different radiosensitivities of tumor cells in oxic and hypoxic regions. Since the delivered curative doses actually lead to local tumor control, they likely overdose the oxic tumor cells while they may be just sufficiently high to inactivate the more resistant hypoxic cells. On the other hand, the subcurative doses may still well exceed the doses required to kill all oxic cells, while they are not sufficient to inactivate all hypoxic cells, which in turn leads to uncontrolled tumors. This excess dose in oxic cells for both dose levels may be the reason for the persistently suppressed proliferative activity, while the resistant hypoxic cells receive curative or subcurative doses that are just slightly above or below the threshold required for complete tumor cell inactivation. It has to be noted, however, that the increased DNA-synthesis in hypoxic cells at the end of the observation period is not predictive for the endpoint local tumor control, since this was observed for both curative as well as subcurative doses. This indicates that an increased proliferative activity within the hypoxic regions does not persist after curative doses while proliferation continues after subcurative doses, at least for some tumor cells. Overall, these findings on the one hand suggest that hypoxic cells are able to resume or maintain DNA synthesis following irradiation, whereas it remains suppressed in oxic cells, exhibiting low BrdU^+^ levels that indicate either effective growth arrest or cell elimination. Consequently, hypoxia confers a relative survival advantage after irradiation across all three treatment modalities. On the other hand the late increase in BrdU^+^/γH2AX^+^ cells observed at 14–21 days after curative irradiation, especially for carbon irradiation, restricted to hypoxic tumor regions, may also suggest persistent or replication-associated DNA damage in a radioresistant hypoxic subpopulation, reflecting delayed repair and/or replication-associated secondary damage [30,31].

As a second tumor subline, the H-tumor was investigated. While inhibition of proliferation as well as an increase with a subsequent decrease in DNA damage was also observed for this tumor, no increase in proliferation at the end of the observation period and no delayed decrease in DNA damage for carbon ions was found in comparison to the HI-tumor. This difference may originate from the fact that these findings were especially pronounced in hypoxic, but not in oxic cells of the HI-tumor, while the H-tumor with its very low hypoxic fraction contains predominantly oxic cells.

Regarding vascular cell response, radiation had minimal impact on tumor vascular structure and perfusion overall. CD31^+^/Hoechs^+^ staining was used to identify perfused, functional vessels, whereas diffusion distance served as a proxy for inter-vessel spacing and perfusion efficiency. The lack of significant differences between HI- and H-tumors across beam modalities, time points and doses indicated robust vascular stability post-irradiation. Carbon ions uniquely promoted a shift from predominantly perfusion-limited hypoxia towards more severe hypoxia subtypes, including cyclic and diffusion-limited hypoxia, when compared to photons and protons [32,33]. In our data, this pattern indicates changes in oxygen distribution and perfusion dynamics that may worsen tumor oxygenation. However, based on the present endpoints, we cannot conclusively distinguish whether these alterations reflect true vascular remodeling at the microvascular level or predominantly functional changes in blood flow and oxygen delivery. The observed predominance of cyclic and diffusion-limited hypoxia after carbon-ion irradiation is consistent with a more chronic and spatially extended hypoxic microenvironment, characterized by recurrent or persistent areas of under-oxygenation. Such conditions are known to favor the selection and maintenance of hypoxia-tolerant tumor cell clones and to promote long-term adaptive processes, including hypoxia-inducible factor (HIF) signaling, metabolic reprogramming, and enrichment of more radioresistant subpopulations. Nonetheless, in the absence of direct measures of vascular remodeling, these implications should be interpreted as plausible, but not definitive, consequences of the hypoxia pattern observed in our study [34]. In such microenvironments, cells can re-establish DNA-synthesis despite prior irradiation. This is consistent with the transient rebound of proliferating BrdU^+^ and BrdU^+^/γ2AX^+^ cells under hypoxia observed at 14 and 21 days post-curative treatment, although the tumors were ultimately cured. As discussed above this late increase in BrdU^+^/γH2AX^+^ cells may reflect persistent or replication-associated DNA damage in a radioresistant hypoxic subpopulation, reflecting delayed repair and/or replication-associated secondary damage, consistent with reports that hypoxia induces transcriptional repression of DNA repair pathways leading to persistent DNA damage and replication-associated secondary damage, although the tumors were ultimately cured [35]. While these observations fit with the established radiobiology of tumor repopulation dynamics [36] in successfully treated tumors, our data do not directly demonstrate effective clonogenic repopulation by the BrdU^+^/γH2AX^+^ cells. Thus, hypoxia-associated tumor repopulation and the potential involvement of cancer stem cells in hypoxic niches should be regarded as plausible, but still hypothetical explanations that cannot be conclusively established from our current data.

Regarding the immune cell response, we found that radiation induces a time-dependent influx of CD68^+^ macrophages into both HI- and H-tumors across all modalities, with modality-specific attenuation and hypoxia-modulated kinetics that point to distinct tumor microenvironment dynamics rather than clearly defined mechanistic pathways. The universal significant increase in total CD68^+^ cells over time is consistent with radiation-induced inflammation and vascular leakage, recruiting monocytes/macrophages to clear debris from dying cells, which is a well-known post-irradiation response. The lesser increase with carbon ions compared to photons or protons suggests a reduced acute inflammatory footprint for high-LET radiation; however, this should be interpreted cautiously, as CD68 does not distinguish between pro-inflammatory (M1-like) and pro-tumorigenic (M2-like) macrophage phenotypes. The delayed increase in macrophage accumulation observed in hypoxic regions, and only at curative radiation doses, suggests that local oxygen tension critically regulates macrophage chemotaxis. In oxic regions, irradiation likely triggers rapid release of damage-associated molecular patterns (DAMPs) and pro-inflammatory cytokines, promoting early macrophage recruitment. In contrast, hypoxic regions show a delayed response, potentially due to impaired perfusion that limits immune cell access or hypoxia-driven metabolic suppression of chemotactic signal production. These are plausible explanations, but they remain speculative in the absence of macrophage subset markers. The clear dose dependence of this effect further supports this interpretation as subcurative doses do not exhibit the same delay, indicating that a threshold level of cytotoxic damage is required for hypoxia to exert a suppressive influence on early macrophage recruitment. At curative doses, the magnitude of tissue injury appears sufficient to amplify hypoxia-associated constraints on signaling and cell trafficking, thereby delaying macrophage infiltration.

Higher baseline CD68^+^ in the HI-tumor after curative carbon ions (oxic) vs. subcurative doses suggest a dose-dependent inflammation. The crossover at 21 d with highest CD68^+^ under hypoxia aligns with the prior observed chronic hypoxia shift under carbon ions. Persistent hypoxic niches could sustain late-stage inflammation, possibly via HIF-driven chemokine release (e.g., SDF-1, CCL2) or tumor cell necrosis releasing attractants during the repopulation rebound. However, without discrimination between M1/M2 subsets, the functional consequences of this late macrophage rebound for tumor control and biological effectiveness remain uncertain. Thus, the late macrophage accumulation under chronic hypoxia should be viewed as a potential contributor to the complex carbon-ion-modified microenvironment, rather than as definitive mechanistic support for increased biological effectiveness.

Radiation triggered a characteristic biphasic response of CD3^+^ T-cell infiltration in HI-tumors, with an initial depletion followed by significant recovery or expansion. In contrast, H-tumors show minimal changes. The initial decrease likely reflects radiation-induced lymphopenia resulting from the direct killing of T cells or impaired trafficking amid acute vascular disruption [37,38]. The subsequent repopulation surge, which is consistent across modalities, dose levels, and oxygen regions, indicates immune reactivation [38]. This reactivation is possibly driven by tumor antigen release induced by massive cell death, which stimulates T-cell homing via chemokines like CXCL9/10 [39].

Similar CD3^+^ T-cell levels and temporal trends in both hypoxic and oxic regions suggest that, unlike macrophages, T cells infiltrate both compartments equally after irradiation. This indicates effective migration into hypoxic niches. In oxic regions, the more pronounced T-cell recovery at curative doses is consistent with increased tumor antigenicity resulting from higher levels of cytotoxic damage. Contrasting responses between tumor models further support this interpretation. HI-tumors display dynamic T-cell repopulation, while H-tumors remain largely unresponsive. This points to greater intrinsic immunogenicity or microenvironmental plasticity in HI-tumors. These characteristics enable adaptive immune engagement and contribute to tumor control, despite proliferative and macrophage rebounds. Overall, curative irradiation induces robust late-phase T-cell reconstitution in HI-tumors independent of radiation modality or hypoxia. However, because CD3 does not distinguish T-cell subsets (e.g., cytotoxic, helper, and regulatory T-cells), the functional nature of this T-cell response and its contribution to the enhanced biological effectiveness of carbon ions cannot be established from our data and should be considered hypothesis-generating rather than mechanistically proven. This highlights the potential for immunotherapy, particularly when combined with particle therapy, but underscores the need for more detailed immune phenotyping in future studies [40].

To the authors’ best knowledge, this is the first comprehensive preclinical study investigating different histological tumor parameters in oxic and hypoxic regions after curative or subcurative doses of low- and high-LET radiation for tumor treatment with known outcome for local tumor control. The results of our study apply primarily to the rat tumor models examined. However, as the underlying response mechanisms in humans are generally considered comparable, and since the most well-known difference in radiosensitivity to humans was adapted using curative and subcurative doses at 100% and 0% TCP, respectively, the results of this study can likely be transferred to humans. Nevertheless, some limitations exist: One is the use of single doses rather than more clinically relevant fractionated doses. A further noteworthy limitation is the limited follow-up time of 3 weeks and the discrete number of observation points. While a longer follow-up time would have been desirable, this was not possible as the tumors shrank to very small volumes as a response to irradiation, which made it impossible to obtain sufficiently large tumor samples. In addition, the first time point was selected at 8 h for practical reasons, which masks the true γH2AX maximum [28]. Finally, the response to proton beams was investigated only for the HI-, but not for the H-tumor for logistic reasons and since the largest differences were expected between photons and carbon ions.

Since neither the dynamics of DNA-synthesis nor DNA damage were predictive for tumor control, it is hypothesized that this decision takes place on the level of very few cells, influenced by cellular signaling, which may additionally interact with the response of vascular and immune cells. In the future, further studies are required to uncover the detailed mechanisms underlying the radiation response of the tumors. As a first step in this direction, a full proteomic analysis on the same tumor samples has been initiated. This may allow us to identify pathways relevant for tumor control, and knowledge of preclinical outcomes together with the results of the present analysis can help to identify the most relevant pathways, which have then to be validated by further prospective experimental studies.

## 5. Conclusions

In this study, we longitudinally analyzed for the first time the temporal pattern of the tumor cell, vascular cell and immune cell responses of prostate carcinomas after treatment with curative or subcurative doses of photons, protons or carbon ions. The analysis included two tumor sublines and special emphasis was put on the impact of oxic and hypoxic microenvironments. While DNA synthesis pattern was independent of radiation modality, dose level and tumor, significant differences were observed between oxic and hypoxic cells. In addition, a delayed decrease in radiation-induced DNA damage was observed after carbon ions vs. photons and protons, and a general delay for all radiation modalities was seen for hypoxic vs. oxic cells. While no general effects on vascular cells were found, a pronounced shift from perfusion-limited towards cyclic and diffusion-limited hypoxia was observed. Finally, a general activation of macrophages was seen, which was dose-dependent for carbon ions in oxic regions. The number of macrophages decreased post irradiation and increased thereafter. For oxic cells, this increase was higher for curative than for subcurative doses. While no direct predictive marker for tumor control could be derived, important insights into the response of different cell compartments and the modulating role of the microenvironmental oxygen conditions were gained. These results together with the available information on local tumor control can guide further studies on the underlying molecular pathways to get a better mechanistic understanding of the key factors leading to local tumor control.

## Data Availability

The datasets presented in this article are not readily available because the high-resolution microscopy data include several TB. Requests to access the datasets should be directed to Ina Kurth.

## Supplementary Materials

The following supporting information can be downloaded at: https://www.mdpi.com/article/10.3390/cancers18162653/s1, Figure S1: Only oxic (Pimonidazole^-^) and BrdU^+^ (top), gH2AX^+^ (middle) and BrdU^+^/ gH2AX^+^ (bottom) cells in the HI-tumor for different beam modalities and for curative (left) and subcurative (right) doses; Figure S2: Only hypoxic (Pimonidazole^+^) and BrdU^+^ (top), gH2AX^+^ (middle) and BrdU^+^/ gH2AX^+^ (bottom) cells in the HI-tumor for different beam modalities and for curative (left) and subcurative (right) doses; Figure S3: Comparison of CD68^+^ (top) and CD3^+^ (bottom) cells in oxic (Pimonidazole^-^) and hypoxic (Pimonidazole^+^) regions in the HI-tumor for curative doses of photons (left), protons (middle) or carbon ions (right); Figure S4: Comparison of CD68^+^ (top) and CD3^+^ (bottom) cells in oxic (Pimonidazole^-^) and hypoxic (Pimonidazole^+^) regions in the HI-tumor for subcurative doses of photons (left) or carbon ions (right); Figure S5: Only oxic (Pimonidazole^-^) and CD68^+^ (top) and CD3+ (bottom) cells in the HI-tumor for different beam modalities and for curative (left) and subcurative (right) doses; Figure S6: Only hypoxic (Pimonidazole^+^) and CD68^+^ (top) and CD3+ (bottom) cells in the HI-tumor for different beam modalities and for curative (left) and subcurative (right) doses.

## Abbreviations

The following abbreviations are used in this manuscript:

γH2AX	Phosphorylated histon protein, DNA damage marker
BrdU	5-bromo-2′-deoxyuridine, proliferation marker
BSA	Bovine Serum Albumin
CBCT	Cone beam computed tomography
CCL2	Chemokine (C-C motif) ligand 2
CD3	Cluster of Differentiation 3-protein complex found on the surface of mature T-lymphocytes (T-cells) that plays a vital role in immune response activation.
CD31	Cluster of Differentiation 31-primarily known as Platelet Endothelial Cell Adhesion Molecule-1 (PECAM-1)
CD68	Cluster of Differentiation 68–glycoprotein found primarily in the lysosomal/endosomal membrane of macrophages, monocytes, and other myeloid cells
CSV	Comma Separated Values (file extension)
CXCL9/10	C-X-C motif chemokine ligand 9 and C-X-C motif chemokine ligand 10-proinflammatory cytokines belonging to the CXC chemokine family that play a key role in recruiting immune cells, particularly T cells, to sites of inflammation
CZI	Data file format for ZEISS microscopy images
DAMP	Damage-Associated Molecular Patterns
DAPI	4′,6-diamidino-2-phenylindole
DCE	Dynamic Contrast-Enhanced
DKFZ	German Cancer Research Center
DNA	Deoxyribonucleic Acid
HIF	Hypoxia-induced factor
HIT	Heidelberg Ion Beam Therapy Center
Hoechst 33342	Perfusion marker
IQR	Interquartile range
LET	Linear Energy Transfer
M2	Alternatively activated macrophages
MRI	Magnetic Resonance Imaging
OMERO	Open Microscopy Environment
PBS	Phosphate-Buffered Saline
PMMA	Polymethylmethacrylate
R3327-AT1	Anaplastic prostate tumor subline
R3327-H	Hormone-dependent prostate tumor subline
R3327-HI	Hormone-independent prostate tumor subline
RBE	Relative Biological Effectiveness
SD	Standard deviation
SDF-1	Stromal cell-derived factor 1
SOBP	Spread-out Bragg peak
TCP	Tumor Control Probability
