# Supplementary material for: Longitudinal Tumor, Vascular, and Immune Cell Response in Two Rat Prostate Carcinomas After Isoeffective Photon, Proton, and Carbon Ion Irradiation: Impact of Linear Energy Transfer, Dose Level, and Hypoxia
Source: Cancers (Basel). 2026 Aug 17;18(16):2653. doi: 10.3390/cancers18162653 (PMC13510923; doi:10.3390/cancers18162653)
Supplement: Supplementary file 1 [file cancers-18-02653-s001.zip › cancers-4422428-supplementary.pdf]

Supplementary materials

Tumor cell response

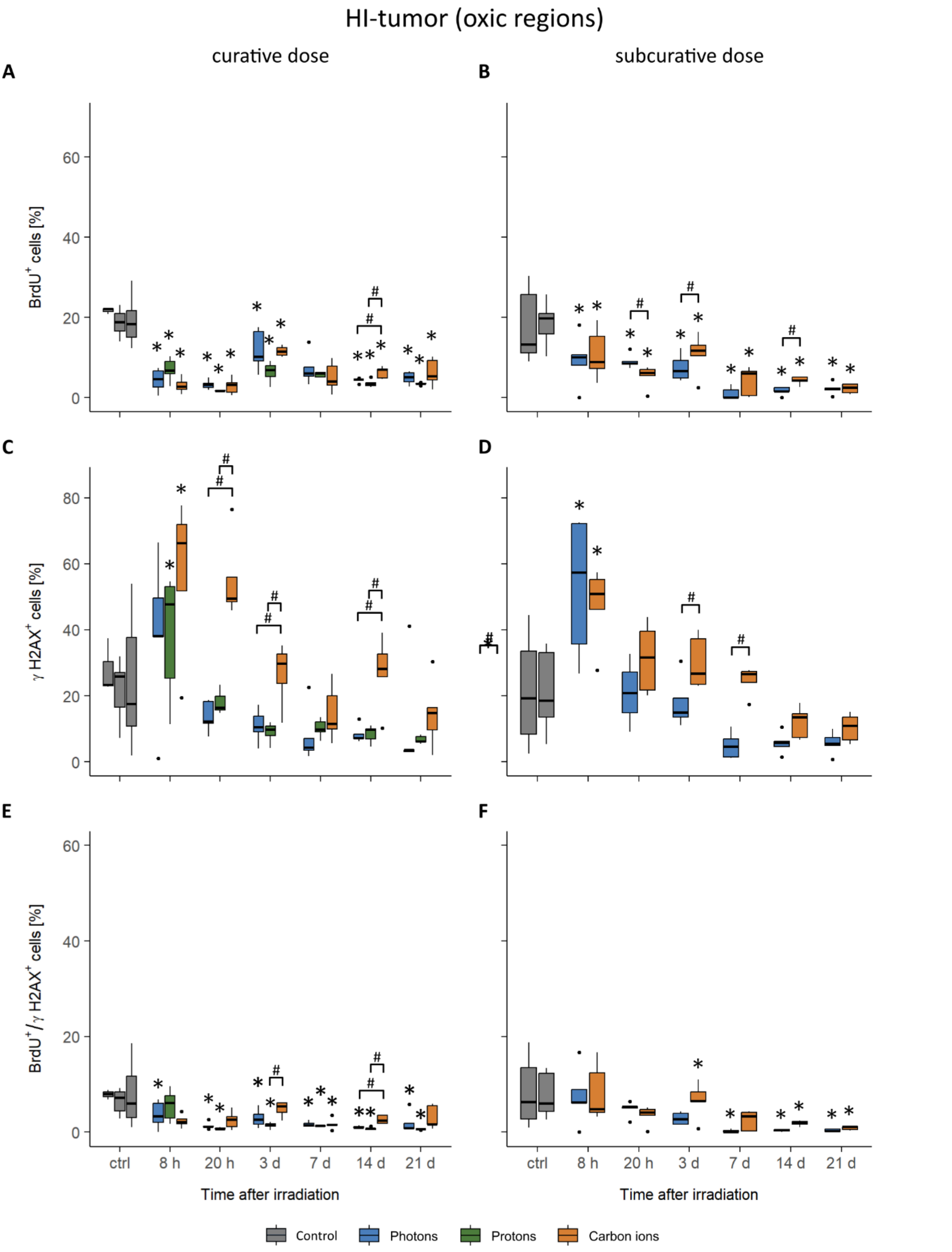

Figure S1. Only oxic (Pimonidazole⁻) and BrdU⁺ (top), γH2AX⁺ (middle) and BrdU⁺/ γH2AX⁺ (bottom) cells in the HI-tumor for different beam modalities and for curative (left) and subcurative (right) doses. Samples were evaluated prior to and at six time points after irradiation. * and # indicate significant differences with respect to controls or between radiation modalities, respectively ($p<0.05$). (Boxplots – median, IQR, whiskers: 1.5 IQR, dots: outliers)

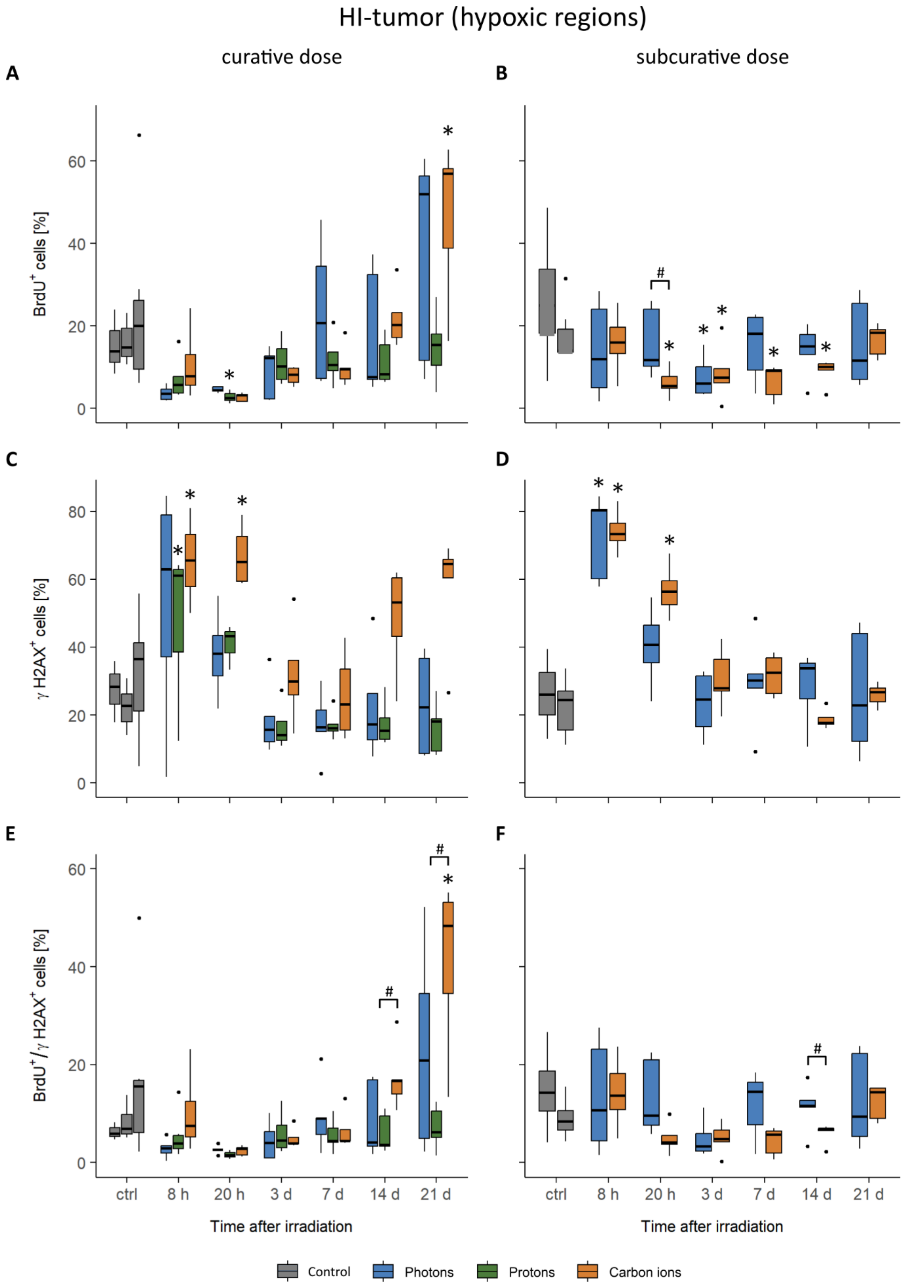

Figure S2. Only hypoxic (Pimonidazole+) and BrdU+ (top), γH2AX+ (middle) and BrdU+/ γH2AX+ (bottom) cells in the HI-tumor for different beam modalities and for curative (left) and subcurative (right) doses. Samples were evaluated prior to and at six time points after irradiation. * and # indicate significant differences with respect to controls or between radiation modalities, respectively ($p<0.05$). (Boxplots – median, IQR, whiskers: 1.5 IQR, dots: outliers)

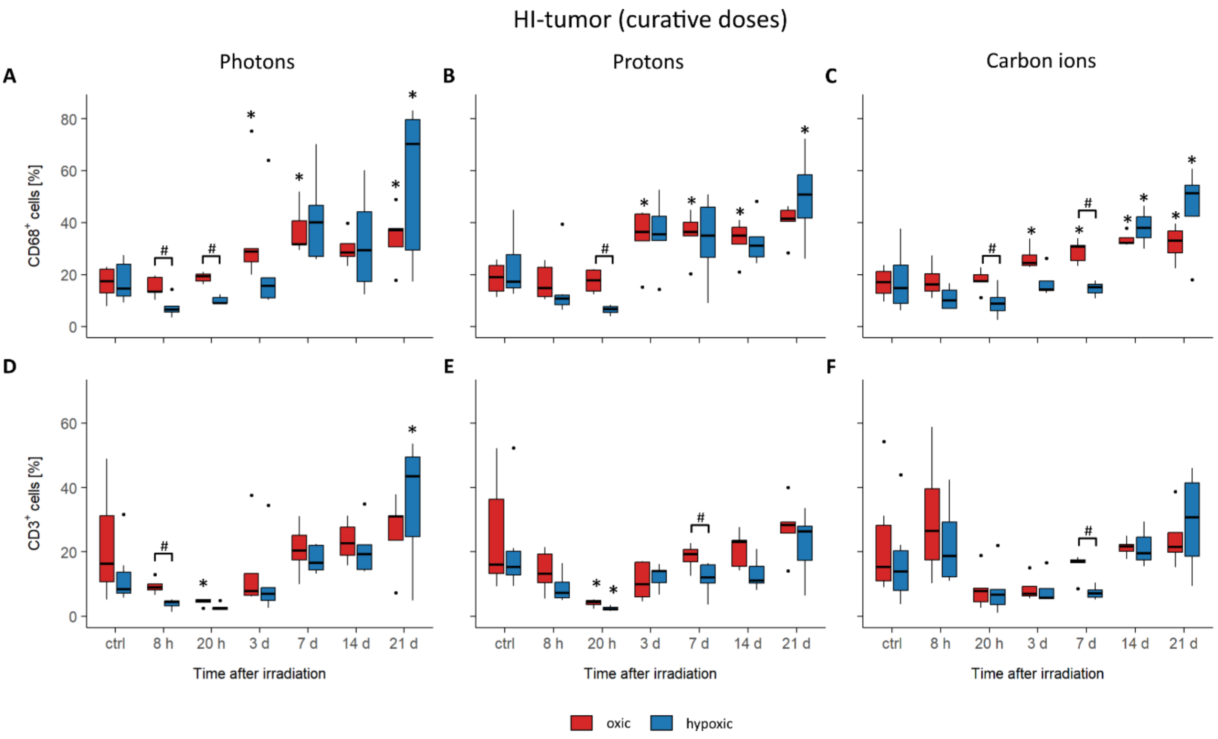

Figure S3. Comparison of CD68$^+$ (top) and CD3$^+$ (bottom) cells in oxic (Pimonidazole$^-$) and hypoxic (Pimonidazole$^+$) regions in the HI-tumor for curative doses of photons (left), protons (middle) or carbon ions (right). Samples were evaluated prior to and at six time points after irradiation. * and # indicate significant differences with respect to controls or between oxygen regions, respectively ($p<0.05$). (Boxplots – median, IQR, whiskers: 1.5 IQR, dots: outliers).

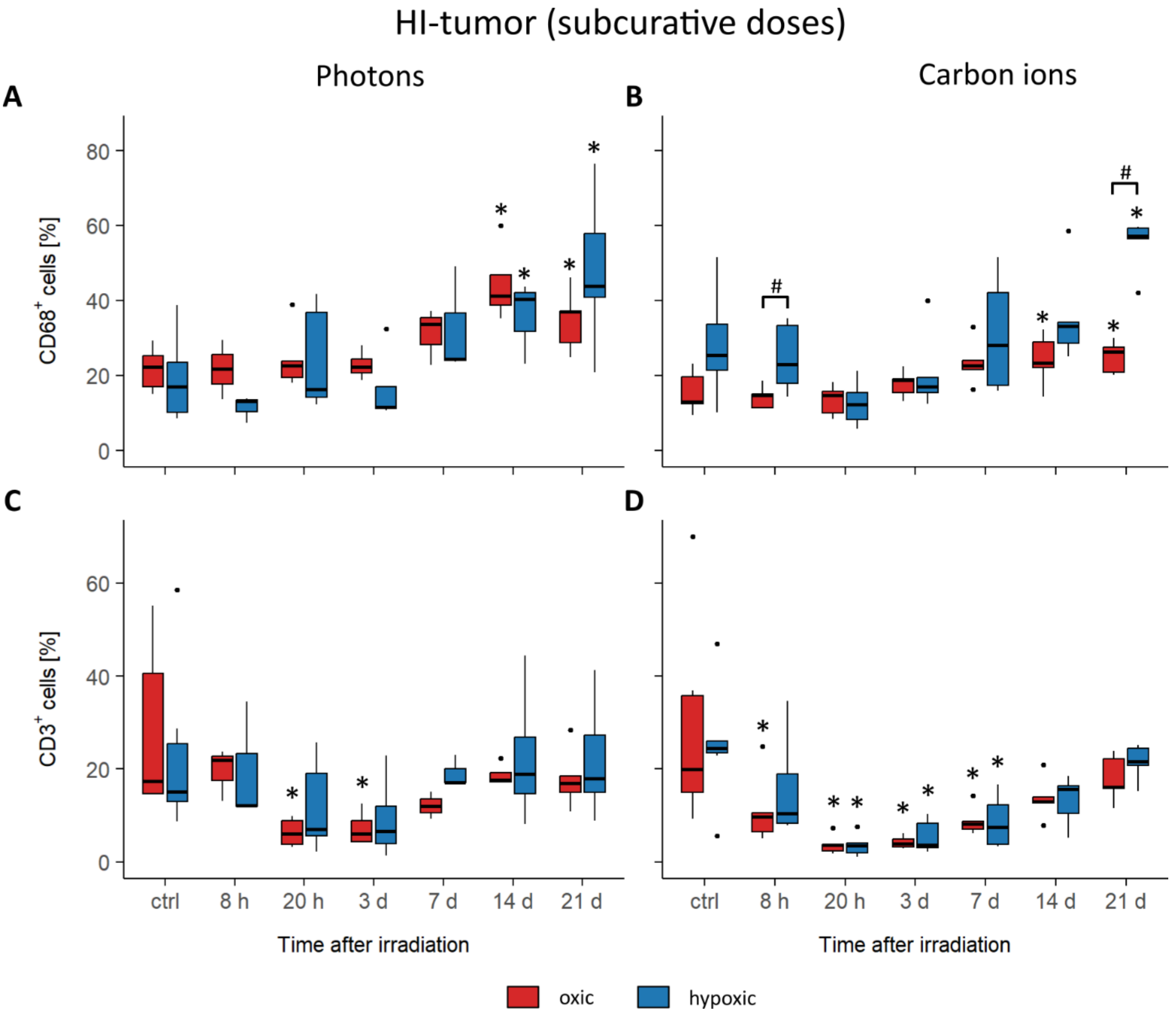

Figure S4. Comparison of CD68+ (top) and CD3+ (bottom) cells in oxic (Pimonidazole-) and hypoxic (Pimonidazole+) regions in the HI-tumor for subcurative doses of photons (left) or carbon ions (right). Samples were evaluated prior to and at six time points after irradiation. * and # indicate significant differences with respect to controls or between oxygen regions, respectively ($p<0.05$). (Boxplots – median, IQR, whiskers: 1.5 IQR, dots: outliers).

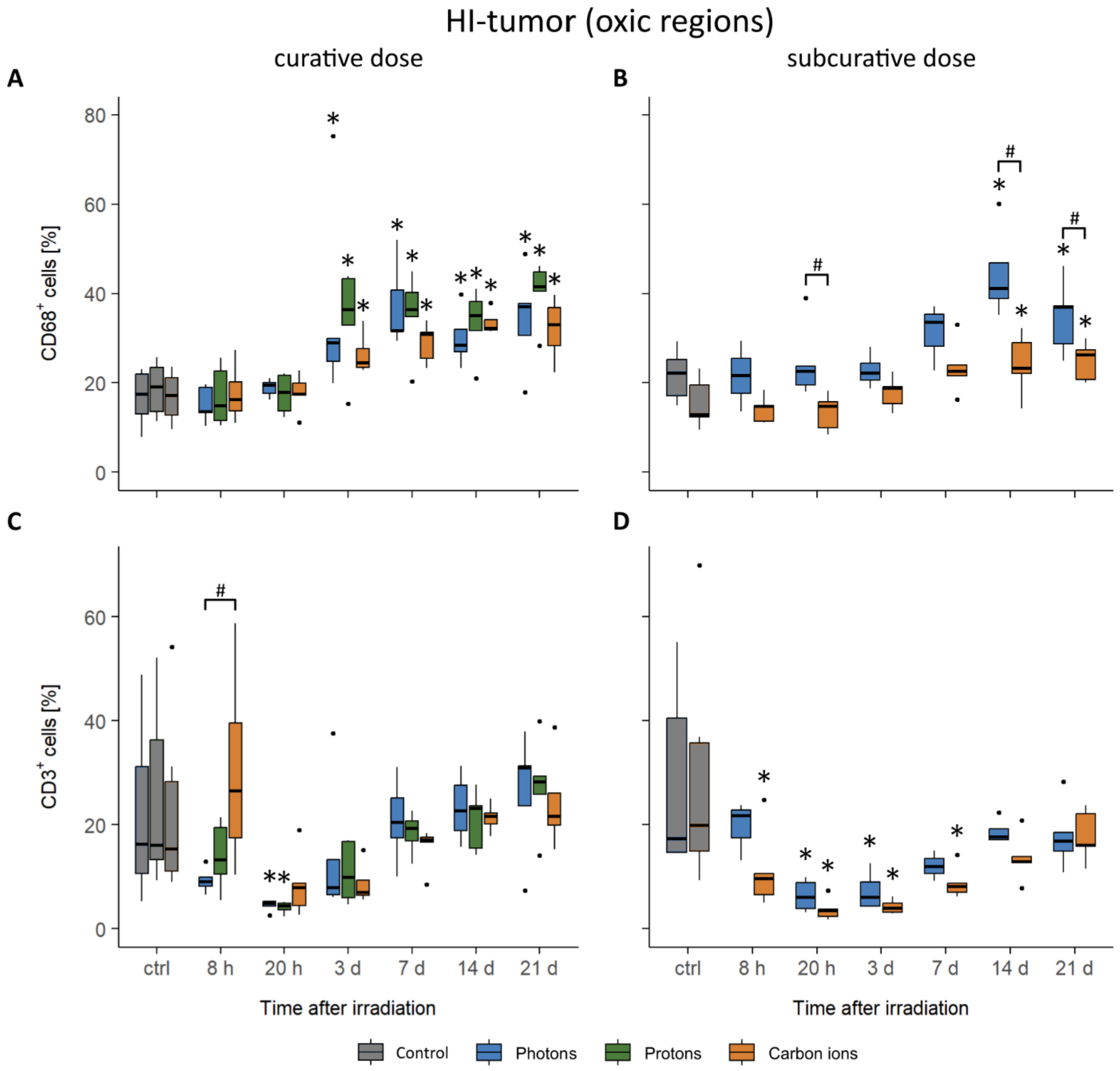

Figure S5. Only oxic (Pimonidazole-) and CD68+ (top) and CD3+ (bottom) cells in the HI-tumor for different beam modalities and for curative (left) and subcurative (right) doses. Samples were evaluated prior to and at six time points after irradiation. * and # indicate significant differences with respect to controls or between radiation modalities, respectively ($p<0.05$). (Boxplots – median, IQR, whiskers: 1.5 IQR, dots: outliers)

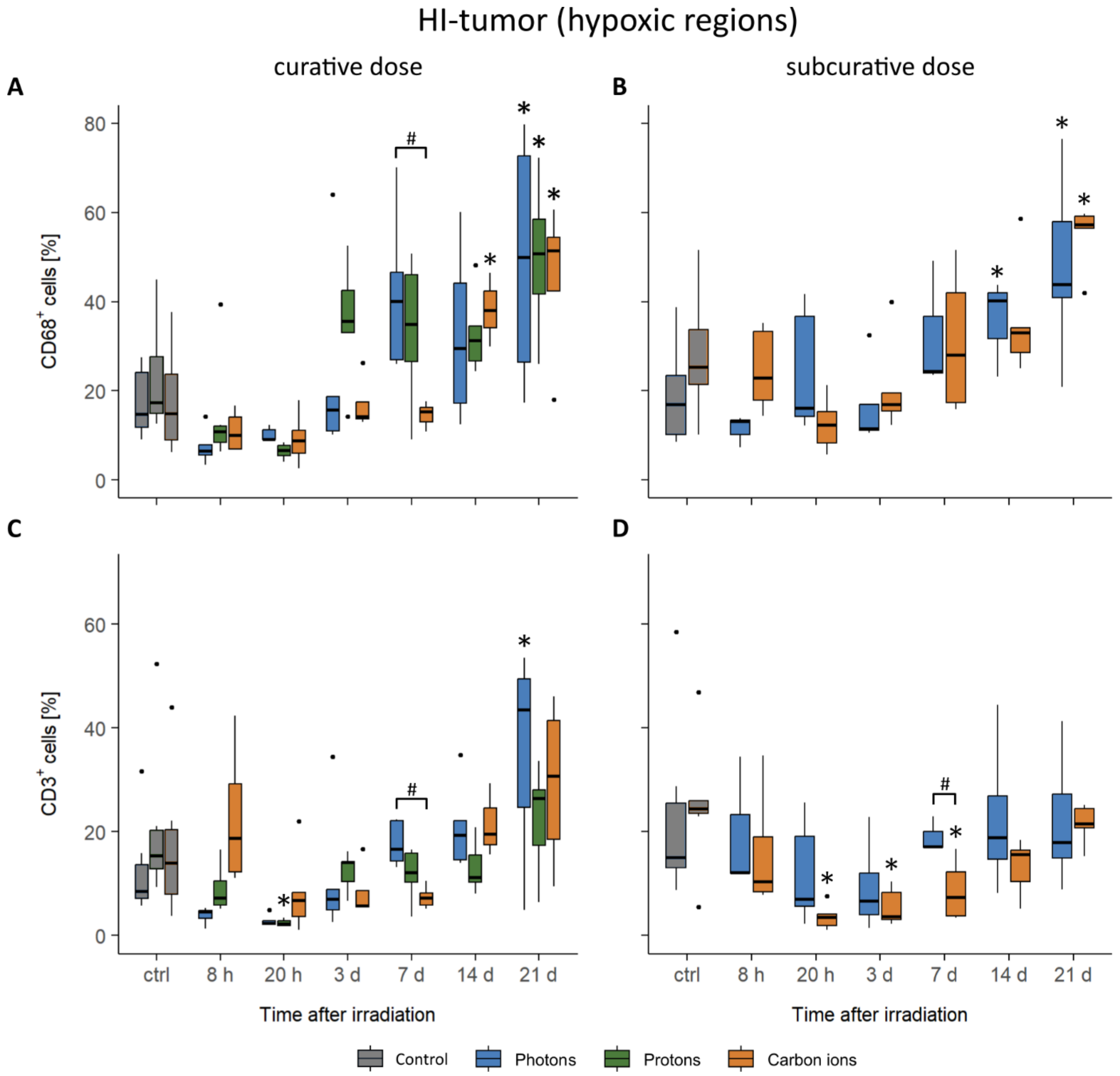

Figure S6. Only hypoxic (Pimonidazole+) and CD68+ (top) and CD3+ (bottom) cells in the HI-tumor for different beam modalities and for curative (left) and subcurative (right) doses. Samples were evaluated prior to and at six time points after irradiation. * and # indicate significant differences with respect to controls or between radiation modalities, respectively ($p<0.05$). (Boxplots – median, IQR, whiskers: 1.5 IQR, dots: outliers)
